# Perinatal risk factors for late neonatal severe acute kidney injury in very low birth weight infants: a retrospective study

**DOI:** 10.3389/fped.2024.1412400

**Published:** 2024-09-30

**Authors:** Hyun Ho Kim, Jihye You, Esther Park, Jin Kyu Kim

**Affiliations:** ^1^ Department of Pediatrics, Jeonbuk National University School of Medicine, Jeonju, Republic of Korea; ^2^ Research Institute of Clinical Medicine of Jeonbuk National University-Biomedical Research Institute of Jeonbuk National University Hospital, Jeonju, Republic of Korea

**Keywords:** acute kidney injury, small for gestational age, intubation, necrotizing enterocolitis, fluid balance, neonate

## Abstract

This study aimed to identify the perinatal risk factors of severe acute kidney injury (AKI) occurring after the first week of birth in very low birth weight (VLBW) infants who survived up to the first week. We conducted a single-center, retrospective cohort study on VLBW infants (birth weight, <1,500 g) delivered at <32 weeks of gestational age (GA) from January 2012 to December 2022. We classified AKI based on changes in serum creatinine and urine output based on the modified The Kidney Disease: improving Global Outcomes (KDIGO) neonatal AKI criteria. Stage 2–3 AKI were considered as severe AKI (sAKI). We performed logistic regression analysis to evaluate risk factors for late neonatal severe AKI identified in the second week after birth. We included 274 VLBW infants. The prevalence of late neonatal severe AKI (sAKI) was 27.4%, with the diagnosis rate of sAKI being higher early after birth. Logistic regression analysis revealed that the factors associated with late neonatal sAKI were small for gestational age (SGA) (OR, 3.02; *P* = 0.032), endotracheal intubation in the delivery room (OR, 2.79; *P* = 0.022), necrotizing enterocolitis (NEC) (OR, 12.41; *P* = 0.029), and decreased minimum weekly fluid balance <0 (OR, 2.97; *P* = 0.012). SGA, intubation in the delivery room, and NEC were associated factors for late neonatal sAKI in VLBW infants. The association of no weekly weight gain with increased late neonatal sAKI risk indicates its use in guiding fluid therapy and aids in biomarker research.

## 1 Introduction

The incidence of acute kidney injury (AKI) in preterm infants within the neonatal intensive care unit (NICU) varies from 18% to 56%, depending on gestational age (GA) (1–4). Because newborns have immature kidney function, body fluids should be maintained through fluid administration and temperature management. During the first week after birth, a physiological weight loss of 10%–20% occurs, accompanied by increased urine output (UO) during the diuretic phase, with rapid changes in body water composition (5, 6). Therefore, AKI in the early postnatal period may show different physiological patterns in neonates. Nephrogenesis begins at 5 weeks of GA and continues until 36 weeks (7). Newborns are susceptible to kidney function impairment due to secondary ischemia caused by hypoperfusion or sudden changes in renal blood flow (8). Furthermore, preterm infants have a higher risk of developing related conditions, such as chronic kidney disease (CKD), later in life, even in the absence of other contributing factors (9).

The definition of neonatal AKI has been standardized through modifications. In 2012, Jetton et al. (10–12) presented the Neonatal Kidney Disease: Improving Global Outcomes (KDIGO) Acute Kidney Injury Classification, which was discussed and established at the 2013 National Institutes of Health workshop and has since been modified for clinical applicability. The neonatal KDIGO diagnostic criteria are based on serum creatinine (sCr) and UO. Initial studies on neonatal AKI used sCr to diagnose AKI, and with the introduction of UO-based criteria for AKI diagnosis, the neonatal modified KDIGO definition remains the gold standard (13). The multinational, multicenter study on critically ill neonates, known as the Assessment of Worldwide AKI Epidemiology in Neonates (AWAKEN), was analyzed based on the neonatal modified KDIGO definition (1, 3, 13–17).

Wu et al. (18) conducted a meta-analysis on AKI in low birth weight infants and found that approximately 25% had AKI. Studies on preterm infants, based on the modified KDIGO definition, showed that AKI was associated with increased mortality and longer hospital stays, and the incidence of AKI increases with lower GA and body weight (19, 20). Additionally, in term infants, congenital heart disease, hypoxic–ischemic encephalopathy (HIE), necrotizing enterocolitis (NEC), and nephrotoxic medication (NTX) were associated with various diseases, including neonatal AKI (21). In preterm infants, neurological damage, such as intraventricular hemorrhage (IVH), hydrocephalus, and HIE, systemic inflammatory responses, such as NEC and sepsis, bronchopulmonary dysplasia (BPD), increased length of hospital stay, and mortality were presented as factors associated with AKI (19, 20). Fluid balance also impacts renal function, and previous studies, including neonates, have reported that high fluid balance is associated with AKI (22).

The timing of diagnosis is primarily determined based on weeks of life after birth. The first week of birth involves physiological changes along with the influence of the mother, and it is presented as a criterion for defining the study population in AKI research (14, 23). In the first week after birth, the mother influences sCr, and because a diuretic phase with increased UO occurs, caution is needed in applying sCr to AKI (13), particularly in cases accompanied by underlying diseases, such as HIE (24). Therefore, different clinical characteristics should be applied for early- and late-onset AKI occurring early after birth (25).

The clinical situation after the first week of birth in preterm infants requires different considerations than before the first week. Risk factors for preterm infants surviving after the first week should be analyzed, but most existing studies focused on AKI in the early postnatal period or are mixed with later postnatal outcomes, making their application as usable factors difficult for clinicians in the NICU. Furthermore, only a few studies focused on neonatal AKI in preterm infants based on UO criteria of the modified KDIGO definition, indicating a need for further research. Therefore, this study aimed to examine the characteristics, associated factors, and risk factors of AKI occurring after the first week of birth in very low birth weight (VLBW) infants who survived the first week.

## 2 Material and methods

### 2.1 Patients

The Institutional Review Board (IRB) of Jeonbuk National University Hospital (JBUH) approved this single-center, retrospective cohort study, with patient consent being waived (IRB No. 2024-03-023). This study adhered to the STROBE reporting guidelines for cohort studies. We included patients admitted to the NICU of JBUH from January 2012 to December 2022, who were born with birth weights <1,500 g and GA of <32 weeks. We excluded those who were not delivered at JBUH, died within 7 days of birth, or were identified with congenital anomalies in the early postnatal period.

### 2.2 Data sources

Demographic and clinical information was collected from electronic medical records. Maternal data included maternal age, duration of premature rupture of membranes, hypertension, diabetes, assisted reproductive technology, and antenatal steroid and antibiotic administration. If early delivery is anticipated, we administer one cycle of antenatal steroids, given twice within a week, to promote fetal lung maturation. If more than a week has passed since the administration of steroids, a second cycle may be considered. Neonatal data include sex, GA, birth weight, small for gestational age (SGA) (26), modes of delivery, Apgar scores at 1 and 5 min, major interventions during neonatal resuscitation performed immediately after birth (27), pH, CO_2_, and base excess from blood gas analysis conducted within the first hour after birth, body temperature at admission, and multiple births. Clinical information available up to the first week included respiratory distress syndrome (RDS), massive pulmonary hemorrhage, air leak syndrome, size of patent ductus arteriosus (PDA) on echocardiography (28), grade of IVH based on the Papile classification (29), and early-onset sepsis with positive cultures before the first week. If a hemodynamically significant PDA is detected during an echocardiogram, we manage it through fluid management to prevent volume overload. If symptoms persist due to the PDA, ibuprofen treatment is considered. We collected sCr and UO during the hospital stay, as well as daily weight measurements, for the diagnosis of AKI. Clinical information available from the second week until discharge included BPD grade based on the revised National Institute of Child Health and Human Development definition (30), pulmonary hypertension requiring medication, PDA size on echocardiography before discharge, NEC of grade II or higher based on the modified Bell's criteria (31), late-onset sepsis with positive cultures after the first week, hydrocephalus, periventricular leukomalacia, stage of retinopathy of prematurity (ROP) (32), length of hospital stay, and death at discharge. During the hospital stay, we recorded the use of antibiotics, steroids, diuretics, caffeine, and vasoactive–inotropic drugs, which are commonly used in the NICU, including nephrotoxic medication (NTX) exposure (33). Fluid balance was calculated based on weight measurements during the hospital stay. The daily fluid balance formula, which calculates the rate of change in daily weight (34), was modified to calculate the weekly fluid balance (WFB) based on weekly weight changes. The modified formula for WFB is as follows: {weekly cumulative weight change (%) = [daily weight (kg)−weight before 7 days (kg)]/weight before 7 days (kg)}.

### 2.3 Definition of AKI

In the NICU of JBUH, sCr was measured on days 1, 3, and 7 after birth, and weekly thereafter. The sCr was calibrated using the Jaffe method (35). UO was measured by weighing diapers every 3 h after changing, and the weight difference was recorded. UO measurements were included from the second day after birth onward due to the impracticality of accurately measuring UO on the first day of admission to the NICU. We defined AKI based on the KDIGO AKI classification (10). Stage 1 AKI is defined as an increase in sCr concentration by ≥0.3 mg/dl within 48 h, an increase of 1.5–1.9 times from the lowest sCr level in the last week (baseline), or when UO is <1 but >0.5 ml/kg/day for 24 h. Stage 2 AKI is defined as an increase in sCr concentration by 2–2.9 times from baseline or when UO is <0.5 but >0.3. Stage 3 AKI is defined as an increase in sCr concentration by ≥3 times from baseline, or when UO is ≤0.3 ml/kg/day. Stage 2–3 AKI were considered as severe AKI (sAKI). The diagnosis of AKI was categorized as early and late sAKI if diagnosed from 24 h after birth up to 7 days of age and from day 8 until discharge, respectively, with the highest stage of AKI being recorded.

### 2.4 Statistical analysis

For the regression analysis, late sAKI was defined as the outcome. Demographic and clinical characteristics, medication use, and fluid balance were compared based on the outcome. Logistic regression analysis was conducted to identify clinical factors associated with late sAKI. Demographic and clinical factors with *P* < 0.05 for a two-sided test were considered statistically significant in relation to the outcome. Subsequently, logistic regression analysis was performed on factors that were amenable to statistical analysis to identify factors associated with late sAKI. Multivariate logistic regression analysis was conducted to statistically ascertain the impact of the extracted risk factors, including diseases, clinical information, and medications that could influence late sAKI. All statistical analyses were performed using R software version 4.3.2 (R Foundation for Statistical Computing, Vienna, Austria).

## 3 Results

### 3.1 Patient demographics and baseline characteristics

**Figure 1:**
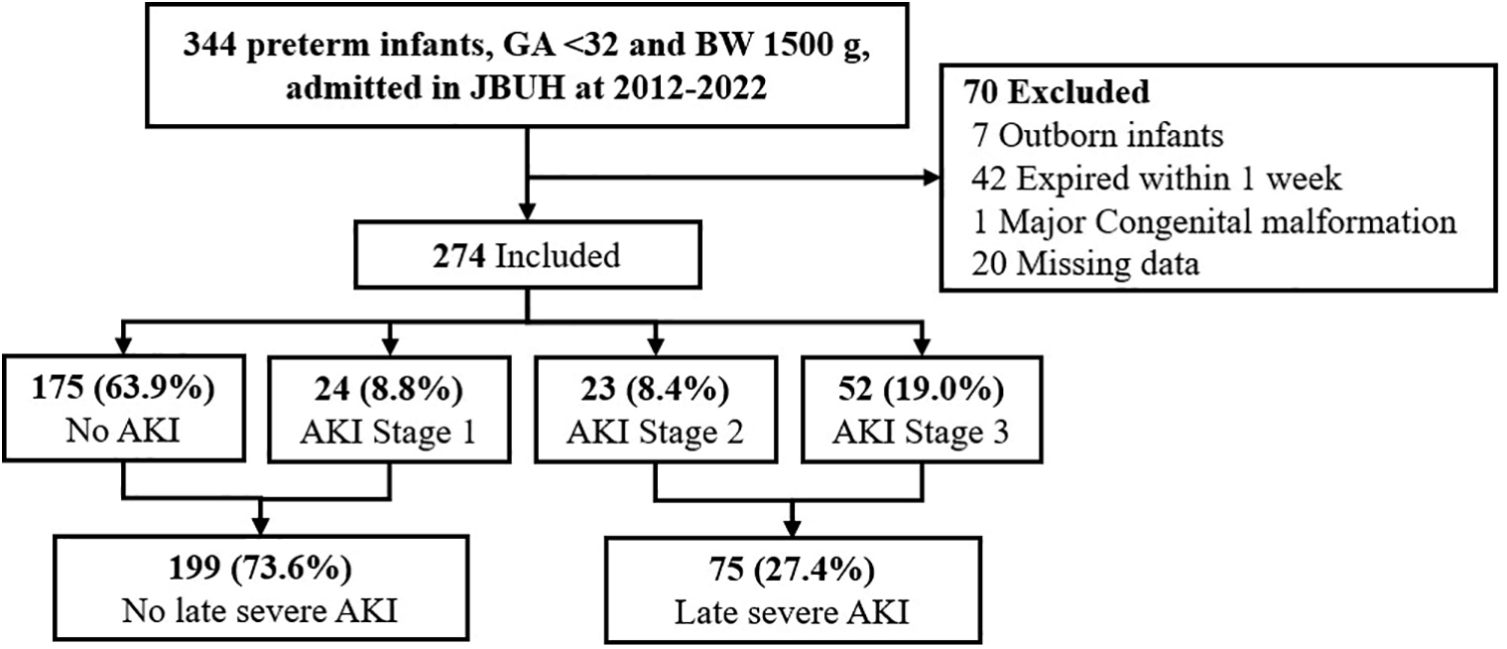
CONSORT flow diagram for the study on severe AKI in preterm infants. This study encompasses all preterm infants with a gestational age of <32 weeks and a birth weight of <1,500 g admitted to JBUH from 2012 to 2022, totaling 344 infants. Among them, 24 infants (8.8%) were diagnosed with Stage 1 AKI, 23 infants (8.4%) with Stage 2 AKI, and 52 infants (19.0%) with Stage 3 AKI.

During the study period, 344 VLBW infants (birth weight < 1,500 g) were delivered at GA <32 weeks. Of these, we excluded 42 who died within the first week, 7 who were transferred from other hospitals after birth, and 1 patient with major congenital anomalies at birth. Finally, 274 patients (119 males and 153 females) met the inclusion criteria for this study. The mean GA and birth weight were 28.06 ± 2.22 weeks and 1,107.74 ± 278.24 g, respectively. AKI stages 1–3 were found in 24 (8.8%), 23 (8.4%), and 52 (19.0%) patients, respectively, with late sAKI confirmed in 75 patients (27.4%). No patients underwent dialysis (Figure 1).

### 3.2 Acute kidney injury incidence and staging

**Figure 2:**
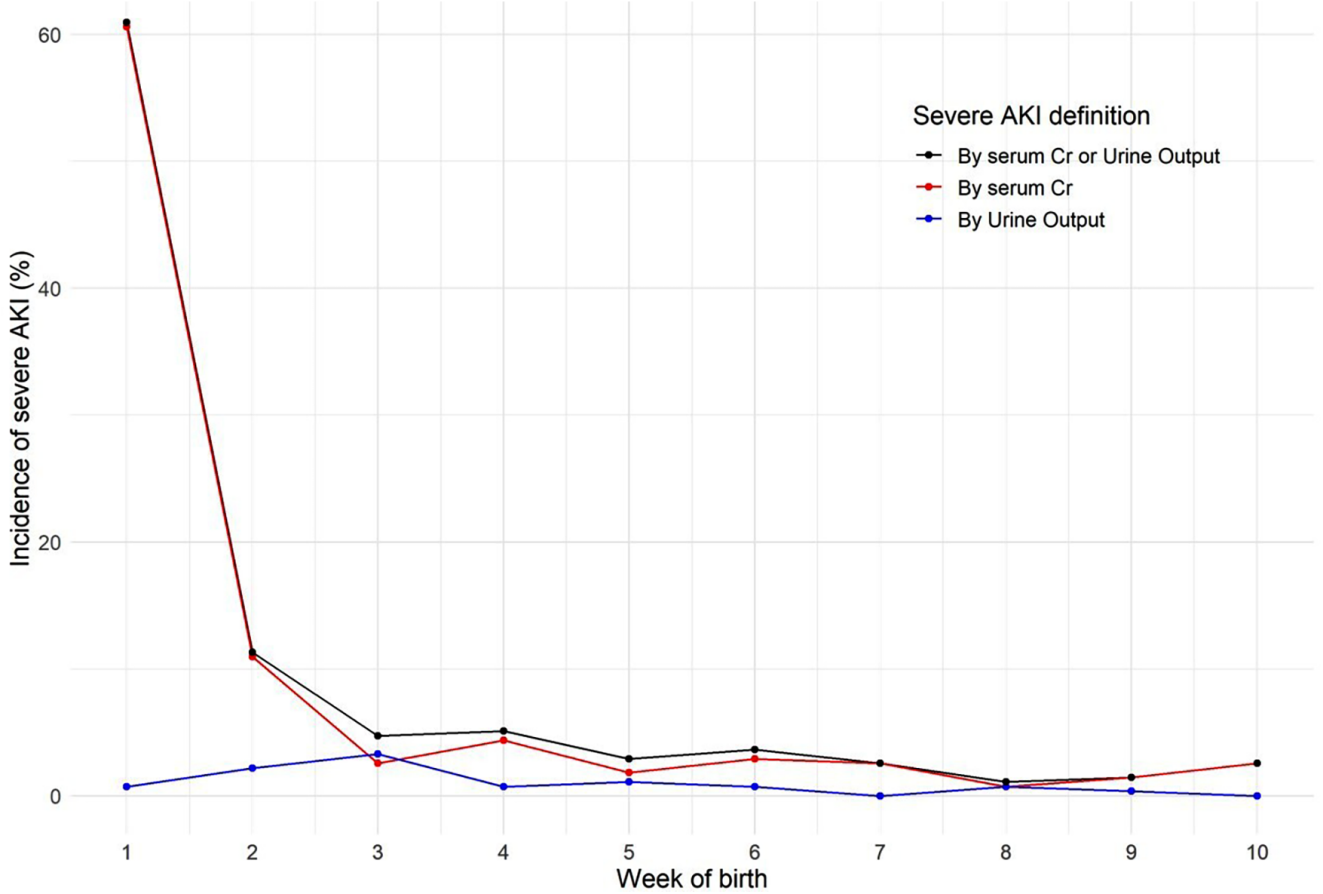
Weekly incidence of severe acute kidney injury (AKI) by diagnostic criteria in preterm infants over 10 weeks. This graph visualizes the percentage of preterm infants diagnosed with severe AKI each week after birth, categorized by different diagnostic criteria: serum creatinine (sCr), urine output (UO), and both combined. A high incidence of AKI was observed in the first week, primarily diagnosed through sCr, with a significant reduction in subsequent weeks.

We confirmed the staging weekly after birth based on the KDIGO AKI diagnostic criteria. In the first week, 167 patients (61.0%) were diagnosed with sAKI, including 166 patients (60.6%) who met the criteria through sCr, and 2 patients (0.8%) through UO. In the second week, 30 patients (11.0%) and 6 patients (2.2%) met the sAKI diagnostic criteria based on sCr and UO, respectively. From the third week, the incidence of sAKI decreased, with 2%–5% of the total confirmed weekly (Figure 2) (Supplementary Table S1).

### 3.3 Maternal and neonatal clinical features associated with late sAKI

We compared the clinical characteristics of the mothers and neonatal clinical features available within the first hour of birth in late sAKI. The late sAKI group had statistically significantly lower GAs and birth weights. The SGA rates, endotracheal intubation during neonatal resuscitation immediately after birth, 1- and 5-min Apgar scores were lower in the late sAKI group compared with those without sAKI (Table 1).

**Table 1:** Comparison of demographic and clinical characteristics.

	All	No late sAKI	Late sAKI	*P*-value*
	(*N* = 274)	(*N* = 199)	(*N* = 75)	
Neonatal factors				
Female	153 (55.8)	111 (55.8)	42 (56.0)	1.000
Gestational age	28.06 (2.22)	28.41 (2.10)	27.13 (2.27)	<0.001
Birth weight	1,106.74 (278.24)	1,164.10 (249.41)	954.57 (294.72)	<0.001
Small for gestational age	26 (9.5)	14 (7.0)	12 (16.0)	0.043
C-section	192 (70.1)	141 (70.9)	51 (68.0)	0.755
Birth resuscitation				
O_2_	249 (91.2)	178 (89.9)	71 (94.7)	0.316
Positive pressure ventilation	204 (74.5)	142 (71.4)	62 (82.7)	0.079
Intubation	187 (68.2)	121 (60.8)	66 (88.0)	<0.001
Chest compression	10 (3.6)	6 (3.0)	4 (5.3)	0.582
Epinephrine	2 (0.7)	1 (0.5)	1 (1.3)	1.000
Apgar score 1 min	5.00 (1.86)	5.30 (1.75)	4.20 (1.94)	<0.001
Apgar score 5 min	7.03 (1.63)	7.27 (1.51)	6.41 (1.78)	<0.001
Blood gas analysis within 1 h				
pH	7.35 (0.11)	7.36 (0.10)	7.34 (0.14)	0.135
CO_2_	39.88 (13.11)	39.32 (12.89)	41.34 (13.64)	0.257
HCO_3_	23.40 (19.66)	23.56 (20.13)	22.97 (18.48)	0.824
Base excess	−4.29 (4.57)	−4.09 (4.18)	−4.83 (5.48)	0.231
Body temperature at admission >36°C	60 (21.9)	45 (22.6)	15 (20.0)	0.762
Multiple births				0.093
Single	172 (63.5)	118 (59.6)	54 (74.0)	
Twin	88 (32.5)	71 (35.9)	17 (23.3)	
Triple	11 (4.1)	9 (4.5)	2 (2.7)	
Maternal factors				
Maternal age	32.65 (4.91)	32.72 (4.92)	32.44 (4.93)	0.671
Premature rupture of membrane ≥18 h	99 (36.1)	73 (36.7)	26 (34.7)	0.866
Duration of premature rupture of membrane	3.25 (7.36)	3.08 (6.98)	3.71 (8.31)	0.526
Maternal hypertension				0.745
No maternal hypertension	217 (79.8)	159 (80.7)	58 (77.3)	
Gestational hypertension	44 (16.2)	31 (15.7)	13 (17.3)	
Chronic hypertension	11 (4.0)	7 (3.6)	4 (5.3)	
Maternal diabetes mellitus				0.884
No diabetes mellitus	246 (89.8)	179 (89.9)	67 (89.3)	
Gestational diabetes mellitus	23 (8.4)	16 (8.0)	7 (9.3)	
Overt diabetes mellitus	5 (1.8)	4 (2.0)	1 (1.3)	
Assisted reproductive technology				0.479
No assisted reproductive technology	188 (68.6)	133 (66.8)	55 (73.3)	
Intrauterine insemination or Ovulation induction	17 (6.2)	12 (6.0)	5 (6.7)	
*In Vitro* Fertilization	69 (25.2)	54 (27.1)	15 (20.0)	
Antenatal steroid				0.280
No	41 (15.0)	29 (14.6)	12 (16.0)	
Before 3 days	158 (57.7)	113 (56.8)	45 (60.0)	
Before 7 days	51 (18.6)	42 (21.1)	9 (12.0)	
After 7 days	24 (8.8)	15 (7.5)	9 (12.0)	
Maternal antibiotics	185 (67.5)	138 (69.3)	47 (62.7)	0.364

Values are number (%) or median (IQR).

* The χ^2^ tests were used to compare proportions of categorical or binary variables. Wilcoxon rank-sum tests used to compare the distribution of continuous variables.

### 3.4 Neonatal diseases and characteristics in the first week

Neonatal diseases and clinical characteristics within the first week of birth were compared based on late sAKI. RDS and IVH of grade ≥1 were higher in the late sAKI group. Moderate to large PDA, identified in the first echocardiogram after birth, was more common in the late sAKI group. AKI stages within the first week of birth based on the occurrence of late sAKI were statistically different (Table 2). The WFB in the first week was −7.51% ± 6.17%, with no statistical difference observed based on late sAKI. Moreover, the proportion of patients receiving ampicillin and cefotaxime >1 day was higher in the late sAKI group compared with the other group (Table 3).

**Table 2:** Clinical characteristics within the first week of life.

	All	No late sAKI	Late sAKI	*P*-value
	(*N* = 274)	(*N* = 199)	(*N* = 75)	
Neonatal disease				
Respiratory distress syndrome	254 (92.7)	179 (89.9)	75 (100.0)	0.010
Pulmonary hemorrhage	4 (1.5)	2 (1.0)	2 (2.7)	0.647
Air leak syndrome	16 (5.8)	9 (4.5)	7 (9.3)	0.220
Patent ductus arteriosus at first echocardiography				0.028
No	124 (45.3)	98 (49.2)	26 (34.7)	
Small	44 (16.1)	35 (17.6)	9 (12.0)	
Moderate	62 (22.6)	39 (19.6)	23 (30.7)	
Large	35 (12.8)	20 (10.1)	15 (20.0)	
No echocardiography	9 (3.3)	7 (3.5)	2 (2.7)	
Intraventricular hemorrhage				0.001
No	205 (74.8)	160 (80.4)	45 (60.0)	
Grade 1	27 (9.9)	16 (8.0)	11 (14.7)	
Grade 2	7 (2.6)	1 (0.5)	6 (8.0)	
Grade 3	18 (6.6)	10 (5.0)	8 (10.7)	
Grade 4	16 (5.8)	11 (5.5)	5 (6.7)	
Expired without cranial sonography	1 (0.4)	1 (0.5)	0 (0.0)	
Early-onset sepsis	7 (2.6)	4 (2.0)	3 (4.0)	0.616
AKI Stage on first week of life				0.009
Stage 0	57 (20.8)	37 (18.6)	20 (26.7)	
Stage 1	50 (18.2)	43 (21.6)	7 (9.3)	
Stage 2	64 (23.4)	52 (26.1)	12 (16.0)	
Stage 3	103 (37.6)	67 (33.7)	36 (48.0)	

Values are number (%) or median (IQR).

The χ^2^ tests were used to compare proportions of categorical or binary variables. Wilcoxon rank-sum tests used to compare the distribution of continuous variables.

**Table 3:** Weekly fluid balance and medication within the first week of life.

	All	No late sAKI	Late sAKI	*P*-value
	(*N* = 274)	(*N* = 199)	(*N* = 75)	
Weekly fluid balance				
Weekly Fluid balance at 1st week, %	−7.51 (6.17)	−7.68 (5.51)	−7.04 (7.67)	0.440
<−20	5 (1.8)	2 (1.0)	3 (4.0)	
−20 to −15	22 (8.0)	17 (8.5)	5 (6.7)	
−15 to −10	68 (24.8)	49 (24.6)	19 (25.3)	
−10 to −5	99 (36.1)	77 (38.7)	22 (29.3)	
−5 to 0	56 (20.4)	39 (19.6)	17 (22.7)	
0–5	15 (5.5)	11 (5.5)	4 (5.3)	
5–10	7 (2.6)	4 (2.0)	3 (4.0)	
>15	2 (0.7)	0 (0.0)	2 (2.7)	
Medication on the first week (≥1 day)				
Ampicillin	224 (81.8)	156 (78.4)	68 (90.7)	0.030
Gentamicin	208 (75.9)	146 (73.4)	62 (82.7)	0.148
Cefotaxime	20 (7.3)	10 (5.0)	10 (13.3)	0.036
Ceftriaxone	3 (1.1)	1 (0.5)	2 (2.7)	0.377
Vancomycin	8 (2.9)	4 (2.0)	4 (5.3)	0.292
Teicoplanin	4 (1.5)	2 (1.0)	2 (2.7)	0.647
Meropenem	10 (3.6)	5 (2.5)	5 (6.7)	0.203
Fluconazole	5 (1.8)	2 (1.0)	3 (4.0)	0.252
Amphotericin B	4 (1.5)	2 (1.0)	2 (2.7)	0.647
Dexamethasone	4 (1.5)	1 (0.5)	3 (4.0)	0.112
Hydrocortisone	84 (30.7)	58 (29.1)	26 (34.7)	0.461
Ibuprofen	236 (86.1)	170 (85.4)	66 (88.0)	0.724
Dopamine	10 (3.6)	8 (4.0)	2 (2.7)	0.864
Dobutamine	2 (0.7)	2 (1.0)	0 (0.0)	0.940
Epinephrine	10 (3.6)	7 (3.5)	3 (4.0)	1.000
Loop diuretics	15 (5.5)	8 (4.0)	7 (9.3)	0.154
Caffeine	218 (79.6)	159 (79.9)	59 (78.7)	0.954

Values are number (%) or median (IQR).

The χ^2^ tests were used to compare proportions of categorical or binary variables. Wilcoxon rank-sum tests used to compare the distribution of continuous variables.

### 3.5 Neonatal diseases and characteristics from the second week until discharge

Neonatal diseases and clinical characteristics from the second week of birth until discharge were investigated based on late sAKI. Grade 3 of BPD or death before 36 weeks was more frequently observed in the late sAKI group. The incidence of pulmonary hypertension diagnosed and treated with medication was also higher in the late sAKI group. The occurrence of NEC stage ≥2 and sepsis after 1 week was higher in the late sAKI group. ROP in stages 2 and 3 was more commonly observed in the late sAKI group. The mortality rate at discharge was 17.3% and 3.0% in the late and non-sAKI groups, respectively. The length of hospital stay was approximately 20 days longer in the late sAKI group compared with the non-late sAKI group (Table 4). The mean WFB from the second week until discharge was 9.80% ± 2.18%, with the late sAKI group approximately 0.7% lower. The maximum and minimum WFB in the late sAKI group was approximately 2.5% higher and 3.5% lower, respectively, compared with the non-late sAKI group. During the hospital stay, most medications were used for longer periods in the late sAKI group. Specifically, antibiotics were prescribed in the late sAKI group for >7 days, such as ampicillin, gentamicin, cefotaxime, vancomycin, and meropenem. Dexamethasone, dopamine, dobutamine, and loop diuretics for >7 days were more likely prescribed in the late sAKI group (Table 5) (Supplementary Table S2).

**Table 4:** Neonatal disease after the second week of life.

	All	No late sAKI	Late sAKI	*P*-value
	(*N* = 274)	(*N* = 199)	(*N* = 75)	
Bronchopulmonary dysplasia				<0.001
No bronchopulmonary dysplasia	158 (57.7)	133 (66.8)	25 (33.3)	
Grade 1	53 (19.3)	38 (19.1)	15 (20.0)	
Grade 2	19 (6.9)	15 (7.5)	4 (5.3)	
Grade 3	25 (9.1)	7 (3.5)	18 (24.0)	
Death at 36th week of corrected age	19 (6.9)	6 (3.0)	13 (17.3)	
Pulmonary hypertension	35 (12.8)	11 (5.5)	24 (32.0)	<0.001
Patent ductus arteriosus on 7 days				0.376
No	197 (71.9)	145 (72.9)	52 (69.3)	
Small	43 (15.7)	32 (16.1)	11 (14.7)	
Moderate	20 (7.3)	11 (5.5)	9 (12.0)	
Large	5 (1.8)	4 (2.0)	1 (1.3)	
Alive without echocardiography	4 (1.5)	4 (2.0)	0 (0.0)	
Expired without echocardiography	5 (1.8)	3 (1.5)	2 (2.7)	
Stage of necrotizing enterocolitis ≥2	8 (2.9)	1 (0.5)	7 (9.3)	0.001
Late-onset sepsis	64 (23.4)	34 (17.1)	30 (40.0)	<0.001
Hydrocephalus				0.148
None	260 (94.9)	191 (96.0)	69 (92.0)	
Yes	9 (3.3)	4 (2.0)	5 (6.7)	
Death without examination	5 (1.8)	4 (2.0)	1 (1.3)	
Periventricular leukomalacia				0.884
None	246 (89.8)	179 (89.9)	67 (89.3)	
Yes	23 (8.4)	16 (8.0)	7 (9.3)	
Death without examination	5 (1.8)	4 (2.0)	1 (1.3)	
Stage of retinopathy of prematurity				<0.001
None	160 (58.4)	129 (64.8)	31 (41.3)	
Stage 1	46 (16.8)	36 (18.1)	10 (13.3)	
Stage 2	33 (12.0)	19 (9.5)	14 (18.7)	
Stage 3	24 (8.8)	10 (5.0)	14 (18.7)	
Death without examination	11 (4.0)	5 (2.5)	6 (8.0)	
Death at discharge	19 (6.9)	6 (3.0)	13 (17.3)	<0.001
Hospital stays	55.26 (26.06)	49.87 (21.45)	69.55 (31.44)	<0.001

Values are number (%) or median (IQR).

The χ^2^ tests were used to compare proportions of categorical or binary variables. Wilcoxon rank-sum tests used to compare the distribution of continuous variables.

**Table 5:** Weekly fluid balance and medication after the second week of life.

	All	No late sAKI	Late sAKI	*P*-value
	(*N* = 274)	(*N* = 199)	(*N* = 75)	
Weekly fluid balance				
The mean value of all weeks,%	9.80 (2.18)	9.98 (2.06)	9.30 (2.43)	0.022
<5%	5 (1.9)	2 (1.0)	3 (4.1)	
5%–10%	143 (53.2)	98 (50.0)	45 (61.6)	
10%–15%	120 (44.6)	95 (48.5)	25 (34.2)	
15%–20%	1 (0.4)	1 (0.5)	0 (0.0)	
>20%	0 (0.0)	0 (0.0)	0 (0.0)	
Maximum value of all weeks	16.35 (5.09)	15.68 (4.20)	18.13 (6.66)	<0.001
<5%	3 (1.1)	1 (0.5)	2 (2.7)	
5%–10%	12 (4.5)	10 (5.1)	2 (2.7)	
10%–15%	97 (36.1)	79 (40.3)	18 (24.7)	
15%–20%	111 (41.3)	81 (41.3)	30 (41.1)	
>20%	46 (17.1)	25 (12.8)	21 (28.8)	
Minimum value of all weeks	3.31 (4.20)	4.23 (3.79)	0.85 (4.26)	<0.001
<−10%	2 (0.7)	1 (0.5)	1 (1.4)	
−10 to −5%	9 (3.3)	3 (1.5)	6 (8.2)	
−5 to 0%	38 (14.1)	14 (7.1)	24 (32.9)	
0%–5%	123 (45.7)	93 (47.4)	30 (41.1)	
5%–10%	91 (33.8)	79 (40.3)	12 (16.4)	
>10%	6 (2.2)	6 (3.1)	0 (0.0)	
Medication after the second week (>7 days)				
Ampicillin	106 (38.7)	56 (28.1)	50 (66.7)	<0.001
Gentamicin	24 (8.8)	7 (3.5)	17 (22.7)	<0.001
Cefotaxime	30 (10.9)	6 (3.0)	24 (32.0)	<0.001
Ceftriaxone	4 (1.5)	1 (0.5)	3 (4.0)	0.112
Vancomycin	28 (10.2)	10 (5.0)	18 (24.0)	<0.001
Teicoplanin	24 (8.8)	13 (6.5)	11 (14.7)	0.06
Meropenem	28 (10.2)	9 (4.5)	19 (25.3)	<0.001
Fluconazole	4 (1.5)	1 (0.5)	3 (4.0)	0.112
Amphotericin B	2 (0.7)	1 (0.5)	1 (1.3)	1
Dexamethasone	25 (9.1)	8 (4.0)	17 (22.7)	<0.001
Hydrocortisone	55 (20.1)	34 (17.1)	21 (28.0)	0.065
Ibuprofen	0 (0.0)	0 (0.0)	0 (0.0)	-
Dopamine	11 (4.0)	1 (0.5)	10 (13.3)	<0.001
Dobutamine	3 (1.1)	0 (0.0)	3 (4.0)	0.029
Epinephrine	1 (0.4)	0 (0.0)	1 (1.3)	0.611
Loop diuretics	16 (5.8)	1 (0.5)	15 (20.0)	<0.001
Caffeine	232 (84.7)	166 (83.4)	66 (88.0)	0.453

Values are number (%) or median (IQR).

The χ^2^ tests were used to compare proportions of categorical or binary variables. Wilcoxon rank-sum tests used to compare the distribution of continuous variables.

### 3.6 Factors associated with late sAKI

Clinical factors associated with late sAKI were extracted for logistic regression analysis to identify clinically meaningful criteria. In the first week, factors included GA, SGA, Apgar scores at 1 and 5 min, intubation at birth, IVH of stage ≥3, early sAKI of stage ≥2, and use of ampicillin and cefotaxime within the first week. From the second week until discharge, we included clinical factors and medication information, including fluid balance information. Clinical factors included pulmonary hypertension, grade 2–3 BPD, or death before the 36th week of corrected age, ROP stage ≥3, NEC stage ≥2, late-onset sepsis, and length of hospital stay. Antibiotics included were ampicillin, gentamicin, cefotaxime, vancomycin, and meropenem used for >7 days. Additionally, the use of dexamethasone, dopamine, and loop diuretics for >7 days was included. WFB criteria that were clinically applicable and statistically significant were established. The analysis included mean WFB <10%, maximum WFB >20%, and minimum WFB <0%. For fluid management, volume depletion is defined as a WFB of <10%, where there is no increase in body weight over a week. Conversely, volume overload is defined as a WFB of >20%, which means the body weight increased by 20% over a week. Multivariate logistic regression analysis results identified SGA and intubation immediately after birth as independent risk factors for late sAKI occurring after the first week. Furthermore, NEC ≥2 was identified as a clinical factor associated with late sAKI. Additionally, a minimum WFB <0%, indicating no weight gain in a week, was associated with developing late sAKI (Table 6).

**Table 6:** Odd ratio for late severe AKI using logistic regression analysis.

Variable	Univariate	*P*-value	Multivariate^a^	*P*-value
	OR (95% CI)		OR (95% CI)	
The first week				
Gestational age	0.78 (0.78–0.69)	0.000		
Small for gestational age	2.54 (2.54–1.12)	0.026	3.02 (1.07–8.27)	0.032
1 min Apgar score	0.72 (0.72–0.62)	0.000		
5 min Apgar score	0.73 (0.73–0.62)	0.000		
Intubation at Birth resuscitation	4.6 (4.6–2.16)	0.000	2.79 (1.21–7.19)	0.022
Moderate to large PDA at first echocardiography	2.44 (1.4–4.24)	0.002		
Intraventricular hemorrhage ≥ stage 3	1.55 (0.72–3.31)	0.260		
Early severe acute kidney injury	1.18 (0.68–2.05)	0.556		
Ampicillin ≥1 days	2.63 (1.12–6.15)	0.026		
Cefotaxime ≥1 days	2.94 (1.17–7.38)	0.022		
From the second week to discharge				
Pulmonary hypertension	8.16 (8.16–3.74)	0.000	2.17 (0.77–5.99)	0.135
Grade 2–3 BPD or death before 36th week of CA	5.16 (5.16–2.81)	0.000		
Retinopathy of prematurity stage ≥3	3.41 (3.41–1.01)	0.049		
Necrotizing enterocolitis stage ≥2	20.58 (20.58–2.49)	0.005	12.41 (1.73–253.07)	0.029
Late-onset sepsis	3.29 (3.29–1.82)	0.000		
Hospital day, days	1.03 (1.02–1.04)	0.000		
Ampicillin >7 days	5.42 (3.02–9.74)	0.000		
Gentamicin >7 days	8.47 (2.6–27.54)	0.000	1.75 (0.91–3.42)	0.098
Cefotaxime >7 days	9 (2.36–34.25)	0.001		
Vancomycin >7 days	3.29 (1.22–8.88)	0.019		
Meropenem >7 days	6.19 (2.23–17.19)	0.000		
Dexamethasone >7 days	4.28 (1.17–15.62)	0.028	2.57 (0.97–6.72)	0.055
Dopamine >7 days	17.38 (2.06–146.99)	0.009	4.7 (1.04–26.19)	0.054
Loop diuretics >7 days	51.05 (6.6–394.85)	0.000		
Mean weekly fluid balance <10%	1.82 (1.04–3.19)	0.036		
Maximum weekly fluid balance >20%	2.8 (1.45–5.41)	0.002		
Minimum weekly fluid balance <0%	7.55 (3.76–15.12)	0.000	2.97(1.27–6.94)	0.012

CA, corrected age; PDA, patent ductus arteriosus; BPD, bronchopulmonary dysplasia.

^a^ Backward elimination used to remove covariates with a *P*-value of >0.2 from the final model.

## 4 Discussion

In this study, we investigated the risk factors for late sAKI in VLBW infants who survived beyond the first week. We found that the prevalence of late sAKI was 27.4%, peaking in the second week and gradually decreasing thereafter. sAKI was primarily diagnosed based on sCr criteria after the second week. Premature infants who require intubation for resuscitation immediately after birth or those who are SGA have an increased risk of developing sAKI after the first week. Furthermore, late sAKI was associated with NEC of stage ≥2, and lack of weight gain in WFB was identified as a risk factor for late sAKI.

Among premature infants who successfully adapted immediately after birth and survived, sAKI was diagnosed in 60% and 27.4% within the first week and the second week until discharge, respectively. AKI stages 1–3 were identified in 8.8%, 8.4%, and 19.0%, respectively. Previous studies showed various incidence distributions depending on the patient group. The AWAKEN study, a representative retrospective study on AKI, involving over 2,000 critical neonates from 24 NICUs across four countries, reported an incidence of approximately 30% depending on the week of birth, with approximately 50% of the patients delivered before 29 weeks being diagnosed with AKI (3). Pantoja-Gomez et al. (36), in a multicenter prospective cohort study focusing on critical neonates and using the neonatal KDIGO classification, found that AKI stages 1–3 were 64.5%, 11.8%, and 23.7%, respectively. A meta-analysis targeting low birth weight infants identified an AKI incidence of approximately 25% (18). Retrospective studies on premature infants showed that the incidence of AKI was 19.5% and up to 60% in infants with VLBW and extremely low birth weight, respectively, indicating that the lower GA, the higher the risk (2, 4). The prevalence in our study was lower compared with other studies, which could be attributed to the exclusion of AKI diagnoses in the first week and the relatively higher mean GA of 29 weeks. A study by Carmody et al. (37), which included a similar patient group to ours, found that 40% of VLBW infants developed AKI, with 16.5% experiencing AKI more than once, which were similar to our study, including stage 1 AKI, with incidence of 36.2%.

At the National Institute of Diabetes and Digestive and Kidney (NIDDK) AKI workshop, the importance of follow-up observation for patients with stage 2 AKI was emphasized to assess the long-term prognosis of neonatal AKI (12). We also monitored the occurrence of AKI weekly, specifically in those with stage 2 AKI. Because we excluded those who died within the first week, approximately 50% of sAKI diagnoses occurred in the first week. By the second week, the sAKI was confirmed at 11.3%, and it remained at 5% until discharge thereafter. Because the occurrence of sAKI was highly likely in the first and second weeks, renal function must be carefully monitored from birth. Additionally, patients diagnosed with sAKI had a hospital stay approximately 20 days longer than those not diagnosed with sAKI. As the length of hospital stay increases, the presence of sAKI based on sCr and UO should be periodically checked.

Our study showed a higher prevalence of AKI compared with studies that diagnosed AKI based on sCr levels (21, 38), which may be due to the influence of AKI diagnosis based on UO. In the early postnatal period, maternal levels influence neonatal creatinine, and the difficulty in obtaining blood samples from neonates posed limitations for the clinical application of AKI diagnosis (10). The neonatal modified KDIGO classification introduced in 2015, which added criteria based on changes in UO, was more easily applied in clinical settings. In our study, we applied separate criteria for AKI based on changes in sCr and UO in preterm infants. In the first week, sAKI was primarily diagnosed based on sCr, and diagnoses based on UO increased from the second week onward, although diagnoses were mainly based on sCr. Nonoliguric AKI was defined as reduced kidney function without decreased UO (39), and most patients diagnosed with sAKI in the first week were classified into the nonoliguric AKI category. UO did not decrease even in sAKI could be due to the diuretic phase accompanying the physiologic weight loss in the early postnatal period. Therefore, nonoliguric AKI without decreased UO should be identified through blood tests within the first week of birth, and further research on nonoliguric AKI within the first week of birth is necessary.

Among the clinical factors identifiable before birth to 1 week after birth, the implementation of endotracheal intubation during resuscitation at the delivery room was identified as a risk factor for sAKI occurring after 1 week. Previous research has found that the Apgar score before 1 week could predict sAKI (40). The Apgar score assesses the condition of the neonate, and endotracheal intubation is decided based on the Apgar score and neonatal resuscitation guidelines in the delivery room. Our results showed that endotracheal intubation was associated with late sAKI than the Apgar score if applied based on the criteria for emergency intervention in clinical practice. Additionally, preterm infants requiring endotracheal intubation immediately after birth likely had issues with cardiopulmonary adaptation or factors related to breathing, such as delivery at an earlier GA or progression to respiratory diseases, such as RDS immediately after birth. In our study, we excluded patients with congenital anomalies, such as heart defects before birth, indicating an association with respiratory diseases, and a high RDS rate in patients with sAKI, similar to a previous study (3). If a preterm infant undergoes endotracheal intubation immediately after birth, preparing for the occurrence of sAKI after surviving the first week is necessary. Nephrogenesis primarily occurs in the latter part of pregnancy, with 60% occurring in the third trimester. Approximately 200,000 nephrons are formed per 1 kg of birth weight (41), and preterm and low birth weight infants alter the final count of nephrons, which is a risk factor for AKI (42). In our study, SGA was considered a potential risk factor even when analyzing preterm infants considering GA, suggesting that SGA may influence nephrogenesis.

Numerous clinical studies have identified infections related to inflammatory responses, with NEC being a prominent related disease (21). Although NEC is a low-frequency disease occurring in <5% of NICU cases, it is highly associated with morbidity and mortality rates of other diseases. Garg et al. (43) conducted a study on 202 patients with NEC and found that sAKI was present in 32.6% of cases. In our study, clinically significant stage ≥2 NEC and sAKI were identified, which was similar to previous research (44–48). The inflammatory response following NEC, potentially leading to a cytokine surge, could be considered a secondary cause, and the infections arising from treatments and interventions for sAKI could also be considered as triggering factors. Because severe diseases, such as NEC, can lead to death, vital signs should be carefully monitored in patients who developed or had sAKI. Pulmonary hypertension in preterm infants is often a secondary condition arising from lung damage due to prolonged mechanical ventilation during NICU stay and is highly associated with BPD. Lung damage not only leads to pulmonary hypertension but also affects overall organ function due to inadequate oxygen supply, including AKI. Previous studies have shown a high association with BPD (2), and our study showed that intractable BPD manifests as pulmonary hypertension. Although the adjusted results did not statistically identify pulmonary hypertension as a significant associative factor with late sAKI, it could be considered for future use as a biomarker to predict AKI. Meanwhile, previous research reported the association between AKI, including within the first week of birth, and BPD. Askenazi et al. (49) reported that stage ≥1 AKI occurring within the first week was associated with BPD or death, and Starr et al. (50), in a study analyzing the AWAKEN cohort, found that AKI occurring within the first month of birth in preterm infants <32 weeks was associated with moderate or severe BPD or death. Future research should also consider the association between BPD and the timing of AKI diagnosis.

Most drugs are excreted through the kidneys and have a high nephrotoxicity in the neonatal period (51, 52). The commonly prescribed NTX in the NICU include acyclovir, amikacin, amphotericin B, gentamicin, indomethacin, piperacillin/tazobactam, and vancomycin, requiring careful monitoring of kidney function (19). A study with the AWAKEN cohort on critically ill neonates during their first week of life found that aminoglycosides were the most frequently used antibiotics, and NTX exposure was associated with AKI. The use of aminoglycosides in conjunction with another NTX resulted in an adjusted hazard ratio of 4.79 (16). Rhone et al. (52) reported that 87% of VLBW infants were exposed to NTX, with gentamicin being the most common drug, and NTX was administered longer in the AKI group. In this study, preterm infants received gentamicin for an average of 3.5 days, the longest duration among NTX drugs. The prospective study Baby NINJA (nephrotoxic injury negated by just-in-time action) targeted critically ill neonates and examined sCr levels at the end of the administration period in patients receiving >3 NTX drugs within 24 h or IV aminoglycosides for >4 days. The results showed that reducing NTX exposure could decrease nephrotic AKI (53). This study assessed the impact based on the standard 7-day prescription guideline for infectious diseases and found a statistically weak association with gentamicin after adjusting for clinical factors. Gentamicin and amikacin, which are mostly associated with proximal tubular damage and nephrotoxicity due to accumulation, require dosage adjustments if renal impairment is suspected (54, 55). Gentamicin, especially, is administered at 36-h intervals in neonates, with a half-life of 5.4–10.0 h (56). Our hospital adjusts drug dosages based on kidney function and typically does not use >3 NTX drugs, which may explain the low association with sAKI. In preterm infants, systemic steroids are considered for poor lung function. Dexamethasone is primarily used to reduce the need for mechanical ventilation or facilitate extubation. Mhanna et al. (57) conducted a retrospective study on a VLBW infants cohort and found that glucocorticoid use was associated with increased systolic BP and decreased prevalence of AKI. In this study, these drugs were mainly prescribed to patients with poor respiratory status, and it is interpreted as a secondary outcome due to respiratory-related effects. Moreover, dopamine was administered to neonates to treat AKI, which may be associated with a higher correlation with sAKI.

In preterm infants, fluid overload early after birth is associated with poor outcomes, including cardiopulmonary diseases, but accurate measurement is challenging (58). Selewski et al. (59), based on the AWAKEN study group, found in their research on late preterm or term neonates that a high fluid balance during the first week of life was associated with the use of mechanical ventilators. Adcock et al. (22), in a retrospective study of 308 individuals, revealed that a high fluid balance was associated with AKI in addition to IVH. Soullance et al. (60), in a study of 191 preterm infants, identified fluid overload at day 10 of life as a risk factor for BPD or death. In preterm infants, fluid balance also affects kidney function measurements, such as sCr. Starr et al. (61) introduced AKI defined by fluid-corrected creatinine, based on the Preterm Erythropoietin Neuroprotection cohort. Only a few studies focused on fluid status after the initial week of birth. After the first week of birth, preterm infants are at a higher risk of dehydration due to their large body surface area and thin epidermis, leading to high insensible water loss (6). Moreover, severe fluid restriction in preterm infants can limit the provision of adequate calories, so clinicians must be aware and strive to achieve fluid balance while considering various factors that can affect the neonate's hydration status (62). However, only a few guidelines exist for proper fluid balance. This study found that the risk of sAKI increases if no weight gain is observed weekly. This analysis differs from the patterns of fluid overload before the first week of birth and is due to many changes occurring before and after the first week of birth.

At the 2017 NIDDK neonatal workshop, the definition of neonatal AKI is evolving and should include future validated markers of structural/organ damage while considering physiological factors. The workshop presented recommendations for research and identified gaps in knowledge (12), and this study was conducted following the guidelines presented. However, this study has several limitations. First, this study was a retrospective, single-institution study conducted on a Korean population, which could introduce bias related to ethnicity and the hospital. Additionally, treatments, such as dialysis or Continuous Renal Replacement Therapy were not considered, which may have limited the treatment options. Based on our results, future research should consider multi-institutional and international collaborative studies. Second, based on the research design, although the causal relationship between clinical factors in the first week and late sAKI was clear, diseases and medications occurring after the second week of birth were only correlated. Future research should consider prospective studies to present causal relationships, considering the timing of AKI. Third, sCr analysis was conducted using the Jaffe method; however, isotope dilution mass spectrometry calibration was not implemented due to the immaturity of the kidneys. Future research may explore such calibration, as has been done in cases of HIE or extreme low birth weight infants (63). Fourth, this study did not address AKI prognosis. Neonatal AKI is known to have a high risk of progressing to CKD, and the KDIGO guideline recommends evaluating for CKD 3 months after an AKI episode (10). Additionally, neonatal AKI also suggests periodic testing for hypertension and albuminuria later in life (64). Therefore, the long-term prognosis after NICU discharge should be verified.

SGA and intubation in the delivery room can be used as associated factors for severe AKI, and consideration for severe AKI is necessary when NEC occurs in VLBW infants. If birth weight does not increase weekly, the risk of late sAKI increases, indicating that this could be used as a guideline for appropriate fluid therapy. Furthermore, the factors associated with late sAKI identified in this study are proposed as evidence that can be used in biomarker research.

## Data Availability

The raw data supporting the conclusions of this article will be made available by the authors, without undue reservation.
