# Supplementary material for: Perinatal risk factors for late neonatal severe acute kidney injury in very low birth weight infants: a retrospective study
Source: Front Pediatr. 2024 Sep 30;12:1412400. doi: 10.3389/fped.2024.1412400 (PMC11471718; doi:10.3389/fped.2024.1412400)
Supplement: Supplementary file 1 [file Table1.pdf]

Supplementary Material

1 Supplementary Tables

Supplementary Table 1. Stage of acute kidney injury from the first week of life to discharge based on serum creatinine and urine output of modified neonatal KDIGO criteria

	AKI by sCr and UO (N = 274)	AKI by sCr (N = 274)	AKI by UO (N = 274)
During the 1st week			
No	57 (20.8)	63 (23.0)	257 (93.8)
Stage 1	50 (18.2)	45 (16.4)	15 (5.5)
Stage 2	64 (23.4)	63 (23.0)	1 (0.4)
Stage 3	103 (37.6)	103 (37.6)	1 (0.4)
During the 2nd week			
No	227 (82.8)	233 (85.0)	259 (94.5)
Stage 1	16 (5.8)	11 (4.0)	9 (3.3)
Stage 2	10 (3.6)	12 (4.4)	1 (0.4)
Stage 3	21 (7.7)	18 (6.6)	5 (1.8)
During the 3rd week			
No	247 (90.1)	262 (95.6)	255 (93.1)
Stage 1	14 (5.1)	5 (1.8)	10 (3.6)
Stage 2	1 (0.4)	0 (0.0)	2 (0.7)
Stage 3	12 (4.4)	7 (2.6)	7 (2.6)
During the 4th week			
No	252 (92.0)	259 (94.5)	262 (95.6)
Stage 1	8 (2.9)	3 (1.1)	10 (3.6)
Stage 2	8 (2.9)	6 (2.2)	2 (0.7)
Stage 3	6 (2.2)	6 (2.2)	0 (0.0)
During the 5th week			
No	257 (93.8)	263 (96.0)	267 (97.4)
Stage 1	9 (3.3)	6 (2.2)	4 (1.5)
Stage 2	5 (1.8)	4 (1.5)	1 (0.4)
Stage 3	3 (1.1)	1 (0.4)	2 (0.7)
During the 6th week			
No	253 (92.3)	261 (95.3)	266 (97.1)
Stage 1	11 (4.0)	5 (1.8)	6 (2.2)
Stage 2	4 (1.5)	2 (0.7)	2 (0.7)
Stage 3	6 (2.2)	6 (2.2)	0 (0.0)
During the 7th week			
No	263 (96.0)	264 (96.4)	273 (99.6)
Stage 1	4 (1.5)	3 (1.1)	1 (0.4)
Stage 2	6 (2.2)	6 (2.2)	0 (0.0)
Stage 3	1 (0.4)	1 (0.4)	0 (0.0)
During the 8th week			
No	263 (96.0)	266 (97.1)	270 (98.5)
Stage 1	8 (2.9)	6 (2.2)	2 (0.7)

Stage 2	1 (0.4)	1 (0.4)	1 (0.4)
Stage 3	2 (0.7)	1 (0.4)	1 (0.4)
During the 9th week			
No	265 (96.7)	267 (97.4)	271 (98.9)
Stage 1	5 (1.8)	3 (1.1)	2 (0.7)
Stage 2	1 (0.4)	1 (0.4)	0 (0.0)
Stage 3	3 (1.1)	3 (1.1)	1 (0.4)
During the 10th week			
No	264 (96.4)	265 (96.7)	273 (99.6)
Stage 1	3 (1.1)	2 (0.7)	1 (0.4)
Stage 2	2 (0.7)	2 (0.7)	0 (0.0)
Stage 3	5 (1.8)	5 (1.8)	0 (0.0)
From 11th week to discharge			
No	252 (92.0)	255 (93.1)	270 (98.5)
Stage 1	7 (2.6)	5 (1.8)	3 (1.1)
Stage 2	4 (1.5)	3 (1.1)	1 (0.4)
Stage 3	11 (4.0)	11 (4.0)	0 (0.0)

Supplementary Table 2. The course of medication administered to the patient during hospitalization

	During 1st week				From 2nd to 4th week				From 5th week to discharge				The entire period of admission				Duration of medication ≥1 day				Duration of medication >3 days			
	All	No late sAKI	Late sAKI	P value	All	No late sAKI	Late sAKI	P value	All	No late sAKI	Late sAKI	P value	All	No late sAKI	Late sAKI	P value	All	No late sAKI	Late sAKI	P value	All	No late sAKI	Late sAKI	P value
	(N = 274)	(N = 199)	(N = 75)		(N = 274)	(N = 199)	(N = 75)		(N = 274)	(N = 199)	(N = 75)		(N = 274)	(N = 199)	(N = 75)		(N = 274)	(N = 199)	(N = 75)		(N = 274)	(N = 199)	(N = 75)	
Ampicillin all, days	4.85 (2.74)	0.61 (2.66)	4.95 (9.89)	<0.001	2.26 (3.90)	1.55 (3.16)	4.13 (4.96)	<0.001	1.80 (5.95)	0.61 (2.66)	4.95 (9.89)	<0.001	8.91 (8.97)	6.72 (6.01)	14.72 (12.37)	<0.001	106 (38.7)	56 (28.1)	50 (66.7)	<0.001	106 (38.7)	93 (36.5)	13 (68.4)	0.012
Gentamicin, days	2.59 (1.83)	2.48 (1.80)	2.87 (1.91)	0.122	0.69 (1.81)	0.45 (1.25)	1.35 (2.70)	<0.001	0.28 (1.30)	0.12 (0.77)	0.71 (2.10)	0.001	3.56 (3.31)	3.05 (2.51)	4.92 (4.57)	<0.001	212 (77.4)	150 (75.4)	62 (82.7)	0.261	24 (8.8)	23 (9.0)	1 (5.3)	0.890
Cefotaxime, days	0.37 (1.48)	0.27 (1.31)	0.63 (1.84)	0.076	0.82 (2.25)	0.53 (1.65)	1.57 (3.26)	0.001	1.26 (4.72)	0.22 (1.49)	4.01 (8.11)	<0.001	2.44 (5.99)	1.02 (2.81)	6.21 (9.57)	<0.001	69 (25.2)	33 (16.6)	36 (48.0)	<0.001	30 (10.9)	26 (10.2)	4 (21.1)	0.280
Ceftriaxone, days	0.04 (0.47)	0.02 (0.21)	0.12 (0.84)	0.103	0.18 (1.39)	0.07 (0.99)	0.45 (2.10)	0.042	0.04 (0.72)	0.00 (0.00)	0.16 (1.39)	0.103	0.26 (2.00)	0.09 (1.01)	0.73 (3.42)	0.016	8 (2.9)	2 (1.0)	6 (8.0)	0.008	4 (1.5)	2 (0.8)	2 (10.5)	0.015
Vancomycin, days	0.09 (0.57)	0.09 (0.62)	0.09 (0.44)	0.919	0.90 (3.02)	0.50 (2.21)	1.96 (4.35)	<0.001	0.91 (3.49)	0.31 (1.55)	2.49 (5.91)	<0.001	1.89 (5.39)	0.89 (3.47)	4.55 (8.08)	<0.001	45 (16.4)	21 (10.6)	24 (32.0)	<0.001	28 (10.2)	23 (9.0)	5 (26.3)	0.045
Teicoplanin, days	0.04 (0.35)	0.02 (0.20)	0.08 (0.59)	0.208	0.88 (2.95)	0.59 (2.03)	1.64 (4.51)	0.008	1.21 (5.50)	0.71 (3.60)	2.53 (8.62)	0.014	2.12 (7.21)	1.32 (4.85)	4.25 (11.08)	0.003	42 (15.3)	25 (12.6)	17 (22.7)	0.06	24 (8.8)	21 (8.2)	3 (15.8)	0.482
Meropenem, days	0.15 (0.84)	0.11 (0.71)	0.25 (1.13)	0.213	0.89 (3.07)	0.41 (1.79)	2.16 (4.88)	<0.001	0.97 (4.92)	0.23 (1.33)	2.93 (8.89)	<0.001	2.01 (6.38)	0.75 (2.38)	5.35 (10.93)	<0.001	49 (17.9)	25 (12.6)	24 (32.0)	<0.001	28 (10.2)	22 (8.6)	6 (31.6)	0.005
Fluconazole, days	0.03 (0.20)	0.01 (0.10)	0.07 (0.34)	0.036	0.22 (1.32)	0.11 (0.84)	0.52 (2.10)	0.02	0.05 (0.48)	0.01 (0.07)	0.19 (0.91)	0.005	0.30 (1.54)	0.12 (0.91)	0.77 (2.49)	0.002	14 (5.1)	5 (2.5)	9 (12.0)	0.004	4 (1.5)	4 (1.6)	0 (0.0)	1.000
Amphotericin B, days	0.01 (0.12)	0.01 (0.10)	0.03 (0.16)	0.308	0.05 (0.56)	0.04 (0.50)	0.09 (0.70)	0.444	0.11 (1.07)	0.07 (0.99)	0.20 (1.24)	0.37	0.17 (1.61)	0.12 (1.49)	0.32 (1.90)	0.351	7 (2.6)	3 (1.5)	4 (5.3)	0.174	2 (0.7)	2 (0.8)	0 (0.0)	1.000
Dexamethasone, days	0.00 (0.00)	0.00 (0.00)	0.00 (0.00)	-	1.77 (4.67)	1.08 (3.28)	3.61 (6.86)	<0.001	0.92 (4.35)	0.34 (2.47)	3.87 (9.59)	<0.001	4.92 (10.80)	0.65 (2.49)	4.63 (9.35)	<0.001	51 (18.6)	18 (9.0)	33 (44.0)	<0.001	25 (9.1)	21 (8.2)	4 (21.1)	0.145
Hydrocortisone, days	1.84 (2.99)	1.73 (2.93)	2.12 (3.13)	0.34	0.79 (2.36)	0.39 (1.66)	1.87 (3.40)	<0.001	1.30 (5.64)	0.26 (1.36)	2.69 (7.77)	<0.001	1.74 (5.60)	3.15 (6.55)	9.60 (16.87)	<0.001	95 (34.7)	63 (31.7)	32 (42.7)	0.118	55 (20.1)	45 (17.6)	10 (52.6)	0.001
Ibuprofen, days	0.86 (0.35)	0.85 (0.35)	0.88 (0.33)	0.584	0.00 (0.00)	0.00 (0.00)	0.00 (0.00)	-	0.01 (0.19)	0.02 (0.21)	0.01 (0.12)	0.946	0.88 (0.40)	0.87 (0.42)	0.89 (0.35)	0.659	236 (86.1)	170 (85.4)	66 (88.0)	0.724	0 (0.0)	0 (0.0)	0 (0.0)	-
Dopamine, days	0.12 (0.71)	0.11 (0.63)	0.13 (0.88)	0.812	0.44 (2.06)	0.12 (1.43)	1.28 (3.03)	<0.001	0.70 (5.05)	0.00 (0.00)	2.56 (9.45)	<0.001	1.26 (6.49)	0.23 (1.86)	3.97 (11.65)	<0.001	39 (14.2)	10 (5.0)	29 (38.7)	<0.001	11 (4.0)	8 (3.1)	3 (15.8)	0.035
Dobutamine, days	0.01 (0.19)	0.02 (0.22)	0.00 (0.00)	0.438	0.05 (0.46)	0.02 (0.16)	0.16 (0.84)	0.02	0.20 (1.59)	0.00 (0.00)	0.72 (2.98)	0.001	0.27 (1.65)	0.04 (0.27)	0.88 (3.06)	<0.001	14 (5.1)	4 (2.0)	10 (13.3)	<0.001	3 (1.1)	1 (0.4)	2 (10.5)	0.003
Epinephrine, days	0.07 (0.50)	0.05 (0.28)	0.13 (0.84)	0.222	0.08 (0.43)	0.01 (0.10)	0.25 (0.77)	<0.001	0.04 (0.22)	0.00 (0.00)	0.13 (0.41)	<0.001	0.19 (0.78)	0.06 (0.30)	0.52 (1.36)	<0.001	25 (9.1)	9 (4.5)	16 (21.3)	<0.001	1 (0.4)	1 (0.4)	0 (0.0)	1.000
Loop diuretics	0.09 (0.48)	0.08 (0.46)	0.15 (0.54)	0.276	0.61 (1.60)	0.18 (0.77)	1.40 (2.29)	<0.001	1.34 (6.94)	0.26 (1.12)	4.21 (12.76)	<0.001	1.95 (7.55)	0.51 (1.51)	5.76 (13.56)	<0.001	69 (25.2)	32 (16.1)	37 (49.3)	<0.001	16 (5.8)	10 (3.9)	6 (31.6)	<0.001
Caffeine	5.60 (3.25)	5.68 (3.26)	5.40 (3.24)	0.528	20.42 (10.46)	14.81 (8.23)	14.84 (8.32)	0.982	16.72 (25.05)	13.15 (19.27)	26.17 (34.58)	<0.001	37.14 (28.95)	33.64 (24.60)	46.41 (36.77)	0.001	238 (86.9)	172 (86.4)	66 (88.0)	0.887	232 (84.7)	217 (85.1)	15 (78.9)	0.698
